# School and Family Environment is Positively Associated with Extracurricular Physical Activity Practice among 8 to 16 Years Old School Boys and Girls

**DOI:** 10.3390/ijerph17155371

**Published:** 2020-07-26

**Authors:** Cristina Romero-Blanco, Alberto Dorado-Suárez, Fabio Jiménez-Zazo, Nuria Castro-Lemus, Susana Aznar

**Affiliations:** 1 PAFS (Physical Activity and Health Promotion) Research Group, Faculty of Nursing, University of Castilla-La Mancha, 13071 Ciudad Real, Spain; cristina.romero@uclm.es; 2 PAFS (Physical Activity and Health Promotion) Research Group, Faculty of Sports Sciences, University of Castilla-La Mancha, 45071 Toledo, Spain; alberto.dorado@uclm.es (A.D.-S.); fabio.jimenez@uclm.es (F.J.-Z.); 3 FENIX (Feminism, Entrepreneurship and Innovation in Exercise) Research Group, Faculty of Sports Sciences, University of Sevilla, 41013 Sevilla, Spain; ncastro@us.es

**Keywords:** adolescent, child, physical activity, school (academic institutions), family

## Abstract

Extracurricular physical activity in children and adolescents can help achieve compliance with the World Health Organization (WHO) recommendations for physical activity. The purpose of this study was to evaluate factors in school and family environments of children and adolescents in Spain that could be related to the practice of extracurricular physical activity. Multistage random cluster sampling was conducted to include 128 schools with the participation of 10,096 students between the ages of 7 and 16. Participants completed the survey of sports habits designed by the National Sports Council. The results revealed a higher participation in extracurricular sports activities among boys (OR: 1.67 (1.5–1.9)) and students in primary education (up to 12 years old) (OR: 1.8 (1.7–2.0)). Likewise, a statistically significant relationship (*p* < 0.005) was observed between families where another family member practiced sports and lower number of screen time hours, improved academic performance, and better self-perceived health. Participation of children and adolescents in extracurricular sports activities seems to be associated with their immediate environment. It is therefore essential to emphasize the importance of establishing physical activity habits from an early age in family and school environments.

## 1. Introduction

Extracurricular activities are very common among children and adolescents. Those related to physical activity can improve health outcomes [1,2] and help meet the World Health Organization’s (WHO) recommendations for physical activity among children and adolescents [3].

The latest report on Spanish children and adolescents [4] shows that physical activity guidelines recommended by the WHO are not being met and most schools do not teach the full physical education teaching load. This situation reduces the possibilities of practicing physical activity (PA) in childhood, which, added to the tendency to participate in more sedentary extracurricular activities, does not help achieve compliance with PA recommendations in this population. At the same time, overweight and obesity levels in children are increasing. In Spain, the prevalence of overweight and obesity in childhood and adolescence is among the highest in Europe, with differences in age, with the highest incidence between 6 and 11 years of age, where boys have a higher prevalence than girls [5]. A consistent finding in the scientific literature is the decline of physical activity during adolescence [6] as well as a lower level of physical activity among girls and adolescent girls compared to boys [7,8].

Participation in extracurricular sports activities has been evaluated as a possible protective factor for overweight among children [9]. In Spain, the number of children involved in sports outside school hours is high, although, once again, there are gender differences (73.3% in boys and 65.6% in girls) [4]. The beneficial effects of extracurricular physical activity are not only limited to improvements in health; in addition, positive academic effects have also been observed [10] as well as reduction of substance abuse in adolescence, particularly in low-income settings [11].

Adherence to physical activity has been inversely related to factors such as age, female sex, perceived effort, and overweight; however, it is necessary to note that we cannot isolate these aspects from socioeconomic and physical contexts of children as well as genetic aspects, which also condition the practice of physical activity [12]. The Spanish Network of Research on Physical Exercise, EXERNET, recommends, among other aspects, that participation in extracurricular activities should be promoted, that sedentary times should be reduced, and that recreation time should be more active. Furthermore, families are encouraged to set an example for children and adolescents [13]. Thus, parent modeling appears to positively influence physical activity among children [14], and even when the child becomes an adolescent, the influence of the family environment on organized activities is paramount [15].

Therefore, this study aimed to investigate the extracurricular activities of children and adolescents and to determine the multiple components of school and family environments that may be related to the practice of extracurricular physical activity.

## 2. Materials and Methods

This study was carried out in Castilla-La Mancha, a region in central Spain comprising five provinces. Multistage random cluster sampling was carried out and adjusted by province. The first units were the educational centers, where the online version of the questionnaire was administered to all students in the randomly assigned class.

The sample was made up of a total of 10,096 schoolchildren, aged between 7 and 16 years old, of whom a total of 5003 were boys and 5093 girls (representing 50.4% girls and 49.6% boys). The study comprised students from public, private, and subsidized schools, equaling a total of 128 participating schools distributed among the different provinces as follows: Toledo (2251 students), Ciudad Real (2704 students), Albacete (2427 students), Cuenca (1460 students), and Guadalajara (1254 students).

Participating schools were recruited by sending invitation letters detailing the purpose of the study. A total of 128 schools agreed to participate and were sent an explanatory letter with instructions for the study procedure.

### 2.1. Questionnaire

The questionnaire used was the one of Sports Habits promoted by the National Sports Council in Spain in 2011.

The questionnaire gathered the following information: sociodemographic variables (age, gender) and information on the extracurricular activities they carried out as well as their parents’ physical activity practice (i.e., students were asked if they carried out any sports or other activities, and they were also asked if their family practiced any type of physical activity). In addition, they were also asked about their academic performance, their activity during recess, their screen time, their means of transport to school, and their self-perceived health.

The variables featured on the questionnaire were dichotomized as Yes/No responses except for the number of hours of screen time (with four options, ranging from less than 2 h to 6 h or more) and academic performance (higher, lower, or equal to that of their peers).

### 2.2. Statistical Analysis

The SPSS 23.0 (IBM Corp, New York, NY, USA) statistical package was used. A descriptive statistical analysis of the variables was performed with the absolute and relative frequency distribution. A bivariate analysis was performed for the different factors related to the practice of extracurricular physical activity using the Pearson’s Chi-square test. Gross odds ratios (OR) were estimated with a 95% confidence interval (95% CI) related to the practice of extracurricular physical activity and educational level. The significance level was set at *p* < 0.005.

## 3. Results

The sample comprised a total of 10,096 students, between the ages of 7 and 16 years old. The students belonged to public, private, and subsidized schools in the Autonomous Community of Castilla-La Mancha, with a total of 128 schools participating in the study.

**Figure 1:**
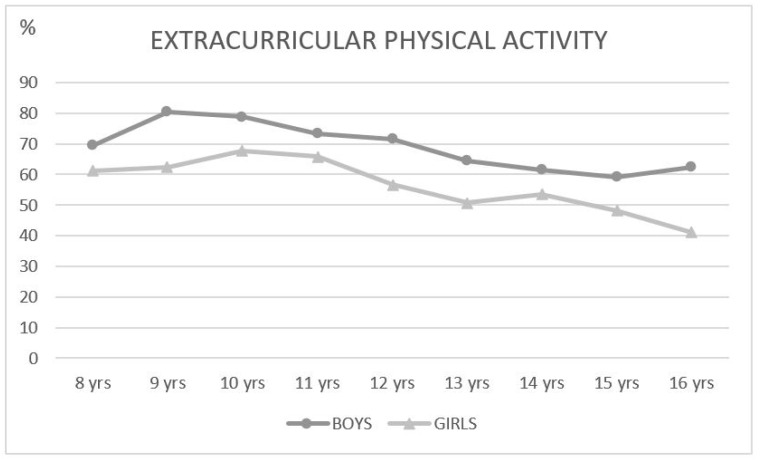
Percentage of students participating in extracurricular physical activity by age and gender. yrs: years.

The practice of extracurricular sports was analyzed in association with gender both on a global level and by age (Figure 1). Statistically significant differences were observed at all ages (except at 8 years old (*p* = 0.054)), where boys showed more after-school physical activity than girls. The greatest increase in physical activity was observed around the age of 9–11 years where nearly 80% of boys practiced sport, dropping to just over 50% at 15–16 years; in the case of girls, the highest percentage of sports practice was at 11 years of age with 65.8% versus 41% at 16 years of age.

Subsequently, the performance of extracurricular physical activity was analyzed by the two educational levels studied: primary education (from 7 to 12 years old) and secondary education (from 12 to 16 years old). Table 1 shows the distribution of participants according to the extracurricular activity carried out. Significant differences were observed both by gender and by educational level. Boys performed more extracurricular sports activities when compared to girls who enrolled in more non-sports related extracurricular activities. Regarding the educational level, a greater link to sports extracurricular activities was observed on behalf of primary school students.

Finally, the factors associated with both the school and the family environments in terms of extracurricular physical activity were studied at both educational levels. Statistically significant differences were found in almost all variables analyzed, both in primary (Table 2) and secondary school (Table 3) students.

When analyzing the relationship between the type of transportation to and from the school and the practice of extracurricular sports, no significant differences were found, i.e., no relationship was found between active transport to school (walking, cycling, using a scooter, etc.) and the practice of sports. This null relationship was verified in terms of both the age of the student and the gender.

For the remaining variables, statistically significant relationships were obtained. Thus, relationships were observed with gender, age, types of activity during recess periods, sport practiced by the family environment (father, mother and siblings), hours of screen time, school performance, and perceived health status. Being a boy and having a physically active family member (father, mother, or sibling) was a statistically significant situation among those enrolled in extracurricular sports. Additionally, being active during recess periods, doing other non-sports extracurricular activities, and spending less than two hours a day screen time were significantly associated with being enrolled in extracurricular sports. Positive associations were also seen in perceived higher academic performance in students who were enrolled in extracurricular sports when compared to their peers; inverse associations were also observed when students considered themselves as not being academically behind their peers.

The analyses were carried out distinguishing by gender, and no changes were observed in the results obtained.

The students who most practiced extracurricular sports activities were males aged between 10–11 years who were also active during recess time. They were living in a family environment where other members also practiced sports, spending less time daily using computers and watching television, with a higher academic performance than their peers, and who considered they were in a better state of health.

## 4. Discussion

This study aimed to evaluate the extracurricular activities of primary and secondary school students and to analyze school and family factors that could be associated with participation in extracurricular physical activities in a sample of 10,096 schoolchildren from Castilla-La Mancha, Spain.

Our findings revealed that the practice of extracurricular physical activity among Spanish students in the region of Castilla-La Mancha was related to the practice of sports activities by their parents, good academic performance, participation in other non-sports extracurricular activities, more active recess time, and less screen time. Furthermore, relationships were found in relation to age and gender, with the practice of extracurricular sports activities being more common in male children around the age of 10.

In this study, we proposed an analysis of extracurricular sports activities according to three main topics; first, the analysis by the two educational levels; second, the analysis by factors related to family environment; and finally, the analyses of factors that could be related to the school environment.

Regarding the first topic, results showed a greater dedication to extracurricular activities at primary education level than at the secondary level. Previous studies have shown a decrease in physical activity levels from childhood to adolescence [7,8,16]; however, to our knowledge, this is the first study that analyzed extracurricular activity participation by the two educational levels. De Meester et al. [17] carried out a study in which they analyzed children’s changes in physical activity during the last year of primary school and at the beginning of secondary school, and one of the reported results was a decrease in extracurricular sports activities at the start of secondary school. In accordance with these findings, in our study, extracurricular sports activity participation seemed to drop by educational level, decreasing in secondary education. However, when assessing the association of remaining variables (school and family) in each of the levels, very similar results were obtained in both groups. Regarding the non-participation in any extracurricular activity, we found that students at secondary education were those who attended fewer activities. In previous studies, higher values of non-participation were reported [18,19], with over 30% of students not participating in extracurricular activities. In our study, different and significant values were found according to the educational level, although these differences were not greater than 30%.

This study highlights the positive correlation of a supportive family environment on the practice of extracurricular sports. Thus, we analyzed whether the father, the mother, or the sibling(s) practiced sports as well as their screen time. Numerous studies have analyzed the influence of the family environment on the activities carried out by children during childhood and adolescence [15,20,21], reporting that having an active family environment encourages children to be active as well. In this study, we observed how all family members (fathers, mothers, and siblings) are relevant in the extracurricular physical activity enrolment. A small effect has been reported in the relationship between parents’ physical activity and children’s physical activity, which also tends to decrease as the child ages; other components may come into play, such as peers’ influences [14]. More recent studies, however, have confirmed this relationship between parents and children, pointing out a strong gender association effect [20]. In these studies, when analyzing the gender effect, a strong relationship was found between father and son, whereas no effect was reported between mother and their sons and/or daughters [14]. In the current study, no gender effect associations were found; thus, it appears that both parents have a positive effect, although it may be slightly more pronounced in the case of the father among secondary school children and the mother in the case of primary school children.

In regard to screen time, considering the study sample participants’ ages, it is up to the parents and therefore the family environment to determine how permissive they are in terms of the number of hours that the children watch television or use a computer, tablet, or video game console. Regarding the time dedicated to screens (television, computer, tablet, etc.), in this study, we observed, especially at the secondary education level, that students who carried out extracurricular physical activity spent less screen time than the ones who did not. The number of screen time hours has been positively related to body mass index (BMI) and adiposity [22,23]; likewise, overweight has been inversely related to the performance of extracurricular sports activities [9]. The current study shows the relationship of the duality between physical activity enrollment and sedentary time and the risk of overweight or obesity in children and young people, observing that students who engaged in sports activities spent less time on television and/or computers.

Other extracurricular activities were also included in the topic dedicated to the family environment, since, as minors, it is the parents who have to sign up and consent (or not) to their children attending to these activities. The benefits of sports practice on academic performance has been demonstrated previously [24], raising the debate as to whether children and adolescents may be enrolled in too many extracurricular activities. The over-programming of activities could lead to the idea that students have less leisure time, and this could have a negative impact on their study time and therefore on their academic performance. However, in this study, those who participated in extracurricular sports were ranked academically equal or higher than their peers and were also enrolled in other non-sports extracurricular activities. These results reaffirm the fact that dedicating time to sports activities does not take time away from studying, but rather it appears to be beneficial. This favorable effect has been observed in both educational levels studied; however, the amount of time dedicated to extracurricular activities was not analyzed in this study, and therefore it is not possible to assess whether there are differences according to their weekly hours spent on these activities.

Finally, we evaluated the effect of certain elements of the school environment on extracurricular physical activity participation. Commuting to school was considered as well as activity time during breaks and perceived academic performance. No statistically significant differences were observed between participating in extracurricular sports activities and active commuting to school. It appears that the factor most strongly associated with active commuting to school is the distance from home to the educational center, [25] although there are also other factors such as the age of the child, the family environment, and other variables concerning the built environment [26]. Having found relationships between family environment and extracurricular sport participation, one would also expect to find relationships with active commuting to school, however, most likely, there are a number of factors (individual, social, political, and environmental) that were not analyzed in this study that, according to previous reports [27,28], may have a strong influence in active commuting.

As for recess time, we asked whether students performed any kind of sport or exercise during these periods or whether they mostly spent their time sitting down and talking to their classmates. Relationships between active recess time and extracurricular sport activity participation were observed in primary school, however, many of the secondary school students did not answer this question, and therefore no clear relationship could be established between this parameter in secondary school students. In a review by Ridgers [29] of physical activity during school recess periods, it was found that boys were more active than girls for all age groups, interpreting that recess time seems to fulfill a need for socialization for girls and an opportunity for competitive play among boys. This review included children aged between 5 and 18 years old. In our study, no differences were observed when analyzing these findings by gender.

Concerning academic performance, students were asked whether their academic results were superior, inferior, or the same as those of their classmates. Both direct and inverse associations were obtained. Thus, students who played extracurricular sports reported to perform better academically than those who did not. This is in line with previous studies where they have associated sports participation with a beneficial effect on academic performance [30].

Finally, we asked about self-perceived health status, which was greater among those who practiced extracurricular sports. The benefits of physical exercise on health are unquestionable [3], and students apparently perceived this based on their responses.

The Spanish regions manage the promotion of school sport in a very different way. In the region of Castilla-La Mancha, teachers receive bonuses for the promotion of sport, thus results may differ from other regions in our country [31].

### Limitations and Future Research

The type of study carried out does not enable the establishment of causal relationships. Thus, solely associations can be observed, although one would expect school and family environments to condition the students’ habits and not vice versa.

This study has a number of limitations. First, the effect of experimental mortality, as some questions were not answered, and more detailed statistical analyses could not be carried out. On the other hand, it would have been interesting to assess the number of hours dedicated to each of the extracurricular activities and to verify whether the results are maintained or whether different effects are observed because of an excess of hours. These data would help us understand the number of hours that is considered most healthy and whether these correspond with the amount of time spent on physical activity recommended by the World Health Organization.

It would be interesting to carry out a study that can also research causal relations. In future research, it would be also interesting to analyze active commuting to school and associated factors.

## 5. Conclusions

In conclusion, the practice of extracurricular sports activities was positively associated with having family members who practiced sports on a regular basis, less use of screens, a perceived high academic performance, and an active recess time. A greater amount of extracurricular sports practice was observed in boys and in primary education. Conversely, no relationship was found in relation to active commuting to school.

The pattern of associations found provides a guide for future efforts towards encouraging greater participation to extracurricular activities that may have an impact on the biopsychosocial health of children and adolescents.

These data, analyzed using a cross-sectional design, show that participation in extracurricular physical activities is favored when the child/adolescent has proactive physical and social environments in both the close family and school. For this reason, it is necessary to develop policies aimed at educating parents that emphasize the importance of helping to establish physical activity habits from an early age. In addition, schools should encourage active recess time and recreation opportunities, especially for girls.

## Figures and Tables

**Table 1:** Distribution of extracurricular classes by educational level.

	Primary Level			Secondary Level			
	BOYS	GIRLS	OR by Gender (Boys: Ref) *	Boys	Girls	OR by Gender (Boys: Ref) *	OR by Level Education (Primaria: Ref) *
**Extracurricular Sport Activities**	1641 (75.8%)	1408 (65.2%)	1.67 (1.5–1.9) *	1801 (64.1%)	1439 (49.3%)	1.84 (1.7–2.0) *	1.8 (1.7–2.0) *
**Extracurricular Non-Sport Activities**	1189 (58%)	1307 (64.1%)	0.77 (0.7–0.9) *	1206 (44.3%)	1572 (55.4%)	0.64 (0.6–0.7) *	1.57 (1.4–1.7) *
**No Extracurricular Activities**	277 (12.8%)	357 (16.5%)	0.74 (0.6–0.9) *	658 (23.4%)	767 (26.3%)	0.86 (0.8–0.9) *	0.52 (0.5–0.6) *

OR: odds ratio, * *p* < 0.005 Ref: reference.

**Table 2:** Relationship between the variables examined and the extracurricular physical activity in primary education (from 7 to 12 years old).

	Physical Extracurricular Activity		
	YES N (%)	NO N (%)	OR 95% CI
**Gender**	3049 (70.5%)	1274 (29.5%)	
Male	1641 (75.8%)	523 (24.2%)	1 (ref)
Female	1408 (65.2%)	751 (34.8%)	1.67 (1.47–1.91) *
**Transport to school center**	3003 (70.7%)	1245 (29.3%)	
Active	1644 (70.1%)	700 (29.9%)	1 (ref)
Not active	1359 (71.4%)	545 (28.6%)	0.94 (0.83–1.08)
**Recess**	2906 (70.1%)	1242 (29.9%)	
Active	1633 (75.8%)	519 (24.2 %)	1 (ref)
Not active	1273 (63.8%)	723 (36.2%)	1.79 (1.56–2.04) *
**Academic performance**	2836 (70.1%)	1208 (29.9%)	
Similar to peers	2087 (70.7%)	863 (29.3%)	1 (ref)
Lower than peers	237 (56.3%)	184 (43.7%)	1.88 (1.53–2.31) *
Higher than peers	512 (76.1%)	161 (23.9%)	0.76 (0.63–0.92) *
**Active father**	2619 (70.1%)	1116 29.9%)	
Yes	1931 (73.3%)	706 (26.7%)	1 (ref)
No	688 (62.7%)	410 (37.3%)	1.63 (1.40–1.89) *
**Active mother**	2537 (69.8%)	1097 (30.2%)	
Yes	1617 (74.8%)	544 (25.2%)	1 (ref)
No	920 (62.5%)	553 (37.5%)	1.79 (1.55–2.06) *
**Active sibling(s)**	2408 (77.6%)	1052 (22.3%)	
Yes	1953 (71.8%)	767 (28.2%)	1 (ref)
No	455 (61.5%)	285 (38.5%)	1.60 (1.35–1.89) *
**Non-Sports extracurricular activities**	2865 (70.1%)	1223 (29.9%)	
Yes	1856 (74.4%)	640 (25.6%)	1 (ref)
No	1009 (63.4%)	583 (36.6%)	1.68 (1.46–1.92) *
**Hours of Television**	2507 (69.7%)	1091 (30.3%)	
Less than 2 h/day	1789 (72.3%)	686 (27.7%)	1 (ref)
From 2 to 4 h/day	523 (64.3%)	291 (35.7%)	1.45 (1.23–1.72) *
From 4 to 6 h/day	114 (62.3%)	69 (37.7%)	1.58 (1.16–2.16) *
Over 6 h/day	81 (64.3%)	45 (35.7%)	1.45 (1.0–2.11) *
**Hours of computer use**	2302 (69.9%)	992 (30.1%)	
Less than 2 h/day	1640 (71.3%)	659 (28.7%)	1 (ref)
Between 2 and 4 h/day	438 (67.9%)	207 (32.1%)	1.18 (0.97–1.42)
Between 4 and 6 h/day	130 (66.0%)	67 (34.0%)	1.28 (0.94–1.75)
Over 6 h/day	94 (61.4%)	59 (38.6%)	1.56 (1.11–2.20) *
**Perceived health**	2818 (70.2%)	1198 (29.8%)	
Excellent, very good or good	2757 (70.7%)	1141 (29.3%)	1 (ref)
Fair or poor	61 (51.7%)	57 (48.3%)	2.26 (1.6–3.3) *

OR: odds ratio, * *p* < 0.005 Ref: reference.

**Table 3:** Relationship between the variables examined and the extracurricular physical activity in secondary education (from 12 to 16 years old).

	Physical Extracurricular Activity		
	YES N (%)	NO N (%)	OR 95% CI
**Gender**	3240 (56.5%)	2491 (43.5%)	
Male	1801 (64.1%)	1009 (35.9%)	1 (ref)
Female	1439 (49.3%)	1482 (50.7%)	1.84 (1.65–2.04) *
**Transport to school center**	3206 (56.4%)	2477 (43.6%)	
Active	1710 (57%)	1290 (43%)	1 (ref)
Not active	1496 (55.8%)	1187 (44.2%)	1.05 (0.95–1.17)
**Recess**	1577 (28.1%)	4037 (71.9%)	
Active	1153 (36.6%)	1995 (63.4%)	1 (ref)
Not active	424 (17.2%)	2042 (82.8%)	2.78 (2.45–3.16) *
**Academic performance**	3088 (55.9%)	2439 (44.1%)	
Similar to peers	1954 (55.8%)	1547 (44.2%)	1 (ref)
Lower than peers	510 (48.9%)	534 (51.1%)	1.32 (1.15–1.52) *
Higher than peers	624 (63.5%)	358 (36.5%)	0.73 (0.63–0.84) *
**Active father**	2762 (55.7%)	2196 (44.3%)	
Yes	1697 (63.1%)	991 (36.9%)	1 (ref)
No	1065 (46.9%)	1205 (53.1%)	1.94 (1.73–2.17) *
**Active mother**	2721 (55.6%)	2174 (44.4%)	
Yes	1410 (62.5%)	845 (37.5%)	1 (ref)
No	1311 (49.7%)	1329 (50.3%)	1.69 (1.51–1.90) *
**Active sibling(s)**	2708 (55.5%)	2170 (44.5%)	
Yes	2088 (58.2%)	1501 (41.8%)	1 (ref)
No	620 (48.1%)	669 (51.9%)	1.50 (1.32–1.71) *
**Non-sports extracurricular activities**	3114 (56.0%)	2450 (44.0%)	
Yes	1712 (61.6%)	1066 (38.4%)	1 (ref)
No	1402 (50.3%)	1384 (49.7%)	1.59 (1.43–1.76) *
**Hours of television**	2737 (56.0%)	2151 (44.0%)	
Less than 2 h/day	1656 (58.6%)	1172 (41.4%)	1 (ref)
From 2 to 4 h/day	828 (54.3%)	697 (45.7%)	1.18 (1.05–1.35) *
From 4 to 6 h/day	180 (50.7%)	175 (49.3%)	1.37 (1.10–1.71) *
Over 6 h/day	73 (40.6%)	107 (59.4%)	2.07 (1.52–2.82) *
**Hours of computer use**	2600 (55.5%)	2084 (44.5%)	
Less than 2 h/day	1386 (59.7%)	936 (40.3%)	1 (ref)
Between 2 and 4 h/day	712 (53.4%)	622 (46.6%)	1.29 (1.13–1.48) *
Between 4 and 6 h/day	295 (51.9%)	273 (48.1%)	1.37 (1.14–1.65) *
Over 6 h/day	207 (45.0%)	253 (55%)	1.81 (1.48–2.21) *
**Perceived health**	3069 (55.8%)	2428 (44.2%)	
Excellent, very good or good	2963 (57.4%)	2199 (42.6%)	1 (ref)
Fair or poor	106 (31.6%)	229 (68.4%)	2.91 (2.3–3.7) *

OR: odds ratio, * *p* < 0.005 Ref: reference.
